# Attitudes, Perceptions and Workplace Factors Predict Well‐Being in Forensic Healthcare Workers Caring for Sex Offenders

**DOI:** 10.1111/jpm.70018

**Published:** 2025-08-02

**Authors:** Chloe Wheeler, Melissa Colloff, Artur Brzozowski

**Affiliations:** ^1^ School of Psychology University of Birmingham Birmingham UK

**Keywords:** attitudes and perceptions, compassion satisfaction, forensic healthcare, sex offenders, workplace well‐being

## Abstract

**Introduction:**

Forensic healthcare workers in mental health settings face significant challenges when working with sex offenders. Balancing staff wellbeing with effective offender rehabilitation remains critical.

**Aim:**

This study examined how attitudes, perceptions and workplace factors influence the wellbeing of frontline forensic healthcare workers caring for sex offenders.

**Method:**

A cross‐sectional design, adhering to the STROBE checklist, was used. Ninety‐one participants from UK privatised mental health settings completed an online survey. Wellbeing was assessed using measures of compassion satisfaction, burnout and secondary traumatic stress, with predictors including attitudes, perceptions and workplace factors.

**Results:**

Positive attitudes, such as understanding offender intent, were associated with improved wellbeing, while excessive trust reduced compassion satisfaction. Perceptions like risk awareness acted as psychological buffers. Workplace factors, including environmental safety and quality supervision, enhanced wellbeing, whereas co‐worker support unexpectedly reduced compassion satisfaction.

**Discussion:**

The findings highlight the importance of rational attitudes in enhancing wellbeing. While punitive perceptions offered emotional buffering, fostering balanced approaches is essential for improving therapeutic environments.

**Limitations and Implications:**

The study focused on privatised UK settings, limiting generalisability to NHS contexts. Future research should explore broader populations.

**Recommendations:**

Employers should enhance environmental safety, supervision and training to foster rational attitudes and address peer dynamics.

**Summary**

- What is known on the subject
  ○ Patient‐facing forensic healthcare workers carving for sex offenders face emotional challenges like burnout and secondary traumatic stress.
  ○ Positive workplace factors, such as safety measures and quality supervision, are linked to better well‐being.
  ○ Attitudes and perceptions of offenders influence both staff well‐being and rehabilitation outcomes.
- What the paper adds to existing knowledge
  ○ This study highlights the distinct effects of attitudes, such as maintaining professional boundaries, on staff well‐being.
  ○ It uncovers unexpected findings, like how co‐worker support can reduce job satisfaction in high‐stress roles.
  ○ It emphasises the role of mental health nurses (MHNs) in supporting healthcare assistants (HCAs), offering insights into their collaborative challenges and needs.
- What are the implications or impacts of the paper
  ○ Promoting rational attitudes, like understanding offender motivations, can enhance staff resilience and improve therapeutic outcomes.
  ○ Employers should prioritise environmental safety and supervision to mitigate burnout and stress among healthcare workers.
  ○ MHNs should lead joint training and mentoring initiatives to equip HCAs with skills that balance therapeutic goals and professional boundaries.
  ○ Training programs must balance fostering protective mechanisms with creating supportive, effective rehabilitation practices.

## 1 Introduction

Forensic healthcare workers directly supporting sex offenders play a pivotal role in maintaining therapeutic environments and ensuring the safety of service users and colleagues in high‐risk and often violent settings. Their work is fundamental to both the rehabilitation of offenders and the broader stability of forensic mental health systems (ve Sorumlulukları and Derleme 2017). Operating at the intersection of care and security, these professionals balance therapeutic engagement with safeguarding demands in environments characterised by aggression, unpredictability and complex mental health needs. Mental health nurses (MHNs), as leaders in these settings, are critical in mentoring frontline, patient‐facing healthcare workers, such as healthcare assistants (HCAs), to ensure effective teamwork and the delivery of safe, high‐quality care.

HCAs play a critical role in daily operations within forensic mental health services. These staff members assist with patients' daily living activities, contribute to the delivery of therapeutic interventions and help manage risk in high‐security environments (Hatcher and Noakes 2010; White et al. 2008). Although they do not hold professional registration, they work closely with qualified professionals, including MHNs, to ensure safe and effective care. Their high levels of patient contact and frontline responsibilities make them a key component of the forensic healthcare team.

Nonetheless, working in the mental healthcare sector also has significant psychological implications (De Hert 2020; Peterson et al. 2008). Healthcare professionals face an increased risk of severe work‐related mental and physical exhaustion, contributing to burnout syndrome—a condition characterised by emotional exhaustion, depersonalisation and reduced personal accomplishment (Maslach et al. 2001). Professionally, it is linked to reduced care quality, high staff turnover and increasing cynicism towards job roles (Scanlan and Still 2019). MHNs play a pivotal role in mitigating burnout among HCAs by providing effective supervision, guidance and support in challenging environments.

Secondary traumatic stress (STS) is also a significant risk for forensic healthcare workers, particularly those exposed to accounts of severe trauma (Stamm 1995; Miller Itay and Turliuc 2023). STS involves symptoms similar to Post Traumatic Stress Disorder, such as intrusive thoughts, avoidance and emotional numbing, stemming from empathetic engagement with traumatised individuals (Hatcher and Noakes 2010). Frontline healthcare workers, often working with distressing and violent service users, are especially vulnerable (Dickinson and Wright 2008). By identifying early signs of STS and promoting evidence‐based coping strategies, MHNs can support the workforce in maintaining their emotional and professional resilience.

While the role is demanding, it is also deeply rewarding, with many frontline workers experiencing high levels of compassion satisfaction. This term refers to the sense of fulfilment and pleasure derived from effectively helping others and making a meaningful difference in their lives. This positive aspect of caregiving supports a sense of role purpose and provides resilience against the emotional toll of working in high‐pressure environments (Hinderer et al. 2014; Stamm 2009). By fostering a sense of purpose and accomplishment, compassion satisfaction can mitigate the risk of burnout and enhance overall wellbeing, contributing to better outcomes for both professionals and service users (Teoh et al. 2023). For MHNs, fostering compassion satisfaction in the workforce can strengthen resilience across teams and improve the therapeutic environment.

### 1.1 Attitudes, Perceptions and Their Influence on Treatment

Attitudes and perceptions shape how individuals interpret and respond to their environments (Pickens 2005). Attitudes involve emotional appraisals, while perceptions are cognitive processes reflecting one's understanding of the world (Cacioppo et al. 1999; Maio et al. 2010). Attitudes are often formed quickly through emotional responses, while perceptions rely on cognitive shortcuts that can lead to biased or stereotypical thinking (Pickens 2005). In the context of working with sex offenders, both attitudes and perceptions can impact professional behaviour and, subsequently, well‐being (Levenson et al. 2007; Higgins and Ireland 2009). This study emphasises attitudes and perceptions as distinct constructs, recognising that perceptions can profoundly affect healthcare workers well‐being, regardless of any negative attitudes they may hold.

Importantly, attitudes and perceptions of healthcare workers can also influence treatment outcomes for sex offenders. Positive attitudes, such as belief in an offender's capacity for change, are essential in fostering a therapeutic alliance, which is critical for successful rehabilitation (Blagden et al. 2013; Harper and Hogue 2015). Conversely, negative perceptions, such as assumptions about dangerousness or immutability, can hinder engagement and contribute to punitive rather than rehabilitative approaches (Mann et al. 2010; Harper and Hogue 2015). These perceptions not only affect offender reintegration but also influence public support for treatment programmes, impacting their implementation and effectiveness (Corabian 2017). Therefore, attitudes and perceptions can shape both healthcare workers' well‐being and treatment outcomes for offenders. Notably, the relationship between these constructs may differ in direction: what benefits treatment outcomes might not necessarily enhance workers' well‐being, highlighting the complexity of balancing these factors in forensic practice.

Education and training play crucial roles in shaping attitudes towards challenging populations. Cognitive psychoeducation has been shown to reduce negative attitudes towards sex offenders among the general public and correctional officers (Kleban and Jeglic 2012; Ware et al. 2012). Individuals without targeted training, such as many frontline forensic healthcare workers, may tend to hold more negative attitudes due to limited access to structured support and resources. This underscores the potential value of structured training in helping these professionals manage biases and build balanced attitudes towards clients, which could ultimately benefit their well‐being and the quality of care they provide (Lea et al. 1999). The current research may help identify factors that promote healthcare workers' well‐being without compromising the effectiveness of treatment outcomes.

### 1.2 Workplace Factors

Workplace factors are also crucial in supporting the well‐being of frontline healthcare staff, particularly those who manage high‐risk populations. These factors closely align with principles of therapeutic security, which involve creating a safe and structured environment to facilitate patient recovery and relational security, which emphasises high‐quality relationships between staff and service users as essential to risk management (Kennedy 2002). Environmental safety (White et al. 2008), quality of supervision (Hatcher and Noakes 2010) and co‐worker support (Mivshek and Schriver 2022) significantly impact job satisfaction, reducing stress‐related outcomes like burnout and STS. For instance, safety provisions help alleviate stress in high‐risk settings, while supervision and peer support offer essential resilience‐building and coping resources. Understanding how these workplace factors impact well‐being among forensic healthcare staff may offer actionable insights for enhancing support mechanisms, ultimately benefiting both staff well‐being and patient care quality. In this study, we examine how these workplace factors influence well‐being among patient‐facing forensic healthcare workers, expanding the understanding of support mechanisms in challenging roles.

### 1.3 Summary of the Background and Research Aims

Balancing the well‐being of frontline forensic healthcare workers with their role in supporting effective treatment outcomes is a critical challenge in this profession, particularly for those working with sex offenders in high‐risk environments. Compassion satisfaction, burnout and STS significantly influence their ability to manage the demands of therapeutic engagement and safeguarding. Despite their essential contributions to offender rehabilitation and forensic mental health systems, the psychological well‐being of patient‐facing forensic staff is under‐researched. In this study, we examine how attitudes, perceptions and workplace factors interact to impact the well‐being of these workers and their capacity to maintain therapeutic relationships. The sample included only staff without attitudinal training, thereby reducing potential confounding effects of such training. By exploring the effects of workplace support and structured training, we aim to identify strategies that promote staff well‐being without compromising treatment effectiveness.

Based on existing research, it was hypothesised that positive workplace factors (such as environmental safety and quality supervision) and rational attitudes towards offenders would be associated with higher compassion satisfaction and lower levels of burnout and STS. Conversely, negative perceptions and attitudes towards offenders were expected to predict poorer well‐being outcomes.

## 2 Methodology

### 2.1 Participants

Participants were patient‐facing forensic healthcare workers (e.g., HCAs, nurses, support workers) employed in private forensic mental health services in the United Kingdom. These services operate alongside NHS facilities and are well established in providing secure care for high‐risk individuals, including those convicted of sexual offences. The sample included staff engaged in the direct care of individuals convicted of sexual offences. Inclusion criteria also specified participants were over the age of 18 and had been working within the service for a minimum of 3 months. Participants who reported receiving training specifically focused on attitudes towards sex offenders were excluded, as were those in administrative roles. While some participants held professional qualifications, none were psychologists or psychiatrists, so all were in frontline roles. Descriptive statistics for the sample, including demographic characteristics and employment details, are presented in the Results section.

### 2.2 Measures

#### 2.2.1 Perception of Sex Offenders Scale

The Perception of Sex Offenders (PSO; Harper and Hogue 2015) is a 20‐item, 6‐point Likert scale designed to measure attitudes towards sex offenders, focusing on punitiveness and stereotypical thinking. The scale demonstrated strong internal consistency and reliability (*α* > 0.92). It includes three subscales: Stereotype Endorsement (*α* = 0.85), which assesses the extent to which participants agree with commonly held negative stereotypes about sex offenders, such as viewing them as irredeemable or inherently dangerous; Sentencing and Management (*α* = 0.92), which evaluates participants' support for punitive measures and restrictive management practices in handling sex offenders; and Risk Perception (*α* = 0.80), which measures participants' beliefs about the likelihood of reoffending or the danger posed by sex offenders. Each item is rated on a scale from 1 (Strongly Disagree) to 6 (Strongly Agree), providing a nuanced understanding of participants' perceptions and attitudes.

#### 2.2.2 Professional Quality of Life Scale—Version 5

The Professional Quality of Life Scale—Version 5 (ProQOL; Stamm 2009) is a 30‐item, 5‐point Likert scale designed to assess both the positive and negative impacts of working in a helping profession. It includes three subscales: Compassion Satisfaction (*α* = 0.88), which measures the pleasure and fulfilment derived from helping others and feeling effective in one's work; Burnout (*α* = 0.75), which assesses feelings of hopelessness, emotional exhaustion and difficulty in achieving professional goals; and STS (*α* = 0.81), which captures the stress and emotional toll of being exposed to others' trauma through one's work. Participants rated each item from 1 (Never) to 5 (Very Often), providing insights into their professional well‐being and potential stressors.

#### 2.2.3 Quality of Work Life Survey

The Quality of Work Life Survey (QWLS; Armstrong and Griffin 2004) evaluates workplace quality of life across eight subscales rated on a 5‐point Likert scale. For this study, three subscales were used: environmental safety, perceived co‐worker support and quality of supervision. Environmental safety (*α* = 0.61) examines perceptions of workplace safety, focusing on the presence of sufficient training, equipment and support to maintain safety among employees. Perceived co‐worker support (*α* = 0.76) reflects the extent to which employees feel they can rely on their colleagues for assistance and maintain positive social networks at work. Quality of supervision (*α* = 0.81) assesses employees' views of their supervisors' competencies, fairness and ability to provide constructive feedback. Participants rated their agreement with items on a scale from 1 (Strongly Disagree) to 5 (Strongly Agree), providing insights into their workplace experiences.

#### 2.2.4 Attitudes Towards Sex Offenders—21

The Attitudes Towards Sex Offenders—21 (ATS‐21; Hogue and Harper 2019) is a refined 21‐item, 5‐point Likert scale designed to measure attitudes towards sex offenders, balancing brevity with strong reliability (*α* = 0.94). It includes three subscales: Trust (*α* = 0.83), which reflects positive attitudes by assessing affective judgements about the trustworthiness of sex offenders and the belief in their potential for change; Intent (*α* = 0.84), another positive attitude subscale, which evaluates rational cognitive explanations of offenders' motivations and behaviours, focusing on thoughtful and balanced attributions about their intent; and Social Distance (*α* = 0.79), a negative attitude subscale, which measures behavioural tendencies to maintain emotional and physical separation from sex offenders, reflecting a desire for detachment or avoidance. Participants rated each item from 0 (Strongly Disagree) to 4 (Strongly Agree), with higher scores indicating stronger attitudes within each subscale.

### 2.3 Procedure

Participants were recruited through a study advertisement accessible via workplace email (following manager authorisation) and social media platforms, including Facebook, WhatsApp and LinkedIn. The snowball method was also employed, encouraging participants to recruit colleagues. This method was selected to maximise reach within secure environments, where direct access to frontline healthcare workers is limited due to confidentiality protocols and shift‐based working patterns. The advertisement included a link to the Qualtrics survey.

Upon accessing the study via the provided link, participants first encountered an information sheet outlining the study's aims, followed by a consent form. Once consent was obtained, participants completed a demographic questionnaire to gather basic personal and employment details. Following this, participants proceeded to complete the four main scales in the following order: PSO, ProQOL, QWLS and ATS‐21. Each scale had clear instructions, and participants could not progress without answering all items, ensuring completeness of data. The estimated survey duration was initially informed by Qualtrics survey predictions and later confirmed by the average completion time recorded in the dataset, which was 18 min.

After completing the questionnaires, participants had the option to provide their email address to enter a raffle, and they were presented with a debrief form that thanked them for their participation, reminded them of their right to withdraw, and provided the researchers' contact information. The raffle was used as an engagement strategy to encourage participation while maintaining voluntary consent. Participants were assured of the confidentiality and anonymity of their responses, identified only by unique participant codes.

Ethical approval for this study was granted by the University of Birmingham STEM Ethical Committee (reference ERN_21‐1594).

### 2.4 Statistical Analysis

#### 2.4.1 Design and Analysis

This quantitative cross‐sectional study adhered to the STROBE (Strengthening the Reporting of Observational Studies in Epidemiology; Appendix A) checklist to ensure transparency and comprehensive reporting. Three standard (enter method) multiple regression analyses were conducted to examine predictive effects among variables. Each ProQOL subscale (compassion satisfaction, burnout and STS) served as the dependent variable in separate multiple regression analyses, using the same set of predictors entered simultaneously in a single step. Predictors included PSO subscales, ATS‐21 subscales and workplace variables (environmental safety, perceived peer support and quality of supervision). Prior to analysis, key parametric assumptions –linearity, normality of residuals, homoscedasticity and multicollinearity–were tested and met. Data were exported from Qualtrics into IBM SPSS Version 27.0 (IBM Corp 2020) for analysis. Incomplete responses (*N* = 40) were removed, leaving 91 participants for analysis.

#### 2.4.2 Power Analysis

Power analysis was conducted using G*Power version 3.1.9.7 (Faul et al. 2007) to determine if the sample size (*N* = 91) was sufficient for nine predictive variables. Using the observed effect sizes for each model (*f* ^2^ = 2.41, 1.18, 1.86) and an adjusted *α* = 0.02 for the three tests, the post hoc power analysis indicated a statistical power of 1, confirming adequate power.

## 3 Results

Participant characteristics are presented in Table 1. This provides an overview of the demographic and professional profiles of the forensic healthcare workers included in the study.

**TABLE 1:** Participant characteristics.

Characteristic	*n*	% (of total *N* = 91)
*Sex*		
Female	43	47.3
Male	46	50.5
Not stated	2	2.2
*Age (years)*		
Range	19–55	
Mean (SD)	30 (9.11)	
*Nationality*		
British	72	79.1%
African	4	4.4%
Asian	8	8.8%
Romanian	3	3.3%
Not stated	4	4.4%
*Length of employment (months)*		
Range	5–132	
Mean (SD)	31.03 (22.16)	
*Training on working with sex offenders*		
Yes (not attitudes training)	13	14.3%
No	78	85.7%
*Professional qualifications*		
Yes (not frontline)	11	12.1%
No	80	87.9%
*Direct work with sex offenders at time of study*		
Yes	91	100%

*Note:* Percentages are calculated based on the total sample (*N* = 91). Where participants chose not to disclose demographic details, this is reported as ‘Not stated’.

The purpose of this study was to examine whether attitudes, perceptions and workplace factors influenced the well‐being of frontline forensic healthcare workers who care for sex offenders and who had not received training specifically focused on attitudes towards this population. Three standard (enter method) multiple regression analyses were conducted to determine the predictive effects on the three components of workplace well‐being: compassion satisfaction, STS and burnout.

### 3.1 Compassion Satisfaction

The first regression model examined whether attitudes and perceptions towards sex offenders, as well as workplace variables, predicted compassion satisfaction. The model was significant (*F* (9, 81) = 21.731, *p* < 0.001) and explained 70% of the variance in compassion satisfaction.

#### 3.1.1 Attitudes

Key findings indicated that several variables significantly predicted compassion satisfaction. Positive attitudes related to the cognitive evaluation of sex offenders (ATS‐21 Intent) were associated with higher levels of compassion satisfaction (*β* = 0.609, *p* < 0.001), suggesting that workers who maintain a balanced, thoughtful approach towards offenders experience greater fulfilment. Similarly, higher scores on social distancing (ATS‐21 Social Distancing), typically considered a negative attitude, also predicted higher compassion satisfaction (*β* = 0.530, *p* < 0.001), indicating that maintaining professional boundaries can enhance satisfaction. Interestingly, higher trust in sex offenders (ATS‐21 Trust), classified as a positive attitude in the hypotheses, was associated with lower compassion satisfaction (*β* = −0.354, *p* = 0.009). This suggests that while trust is generally constructive, excessive trust may undermine well‐being.

#### 3.1.2 Workplace Factors

In terms of workplace factors, environmental safety had a strong positive impact on compassion satisfaction (*β* = 1.190, *p* < 0.001), underscoring the role of a secure environment in enhancing job satisfaction. Perceived co‐worker support negatively predicted compassion satisfaction (*β* = −0.484, *p* = 0.038), indicating that reliance on co‐workers may detract from job satisfaction in this context (Table 2).

**TABLE 2:** Psychological and workplace variables predict compassion satisfaction.

Variable	Unstandardized coefficients		Standardised coefficients beta	Sig.	95% CI	
	*B*	Std. error			Lower bound	Upper bound
*PSOS*						
Sentencing Management	0.143	0.095	0.247	0.134	−0.045	0.332
Stereotype Endorsement	0.138	0.136	0.100	0.314	−0.133	0.409
Risk Perception	−0.058	0.142	−0.038	0.684	−0.340	0.224
*ATS‐21*						
Trust	−0.354	0.131	−0.288	0.009*	−0.616	−0.093
Social Distancing	0.530	0.160	0.395	0.001*	0.211	0.849
Intent	0.609	0.146	0.597	< 0.001*	0.318	0.900
*QWLS*						
Environmental Safety	1.190	0.183	0.612	< 0.001*	0.826	1.555
Quality of Supervision	0.223	0.133	0.144	0.097	−0.041	0.488
Co‐worker Support	−0.484	0.229	−0.208	0.038*	−0.940	−0.029

*Note:* Multiple Regression analysing the following predictors: Perception of Sex Offenders Scale (PSOS), Attitudes Towards Sex Offenders (ATS‐21), Quality of Work Life Survey (QWLS).

* *p* < 0.05.

### 3.2 Secondary Traumatic Stress

The second regression model focused on STS. This model was significant (*F* (9, 81) = 10.645, *p* < 0.001) and explained 55% of the variance in STS.

#### 3.2.1 Perceptions and Attitudes

Several predictors were significantly associated with STS. Negative perceptions of sex offenders, specifically the belief that they should receive harsher sentences (PSO Sentencing Management), were linked to lower levels of STS (*β* = −0.273, *p* = 0.012). This counterintuitive finding suggests that holding more punitive views reduces the emotional impact of working with this population. Similarly, risk perception (PSO Risk Perception) negatively predicted STS (*β* = −0.441, *p* = 0.007), implying that healthcare workers who perceive sex offenders as high‐risk individuals experience less emotional strain. Additionally, cognitive evaluations of sex offenders (ATS‐21 Intent) predicted significantly lower levels of STS (*β* = −0.950, *p* < 0.001), highlighting that a rational and balanced perspective on offenders reduces emotional stress.

#### 3.2.2 Workplace Factors

Workplace variables also played a crucial role. Environmental safety was a significant negative predictor of STS (*β* = −0.659, *p* = 0.002), suggesting that safer work environments alleviate STS (Table 3).

**TABLE 3:** Psychological and workplace variables predict secondary traumatic stress.

Variable	Unstandardized coefficients		Standardised coefficients beta	Sig.	95% CI	
	B	Std. error			Lower bound	Upper bound
*PSOS*						
Sentencing Management	−0.273	0.106	−0.527	0.012*	−0.483	−0.063
Stereotype Endorsement	0.132	0.152	0.107	0.387	−0.170	0.435
Risk Perception	−0.441	0.158	−0.323	0.007*	−0.755	−0.126
*ATS‐21*						
Trust	0.123	0.147	0.112	0.405	−0.169	0.414
Social Distancing	0.073	0.179	0.061	0.685	−0.283	0.429
Intent	−0.950	0.163	−1.043	< 0.001*	−1.275	−0.626
*QWLS*						
Environmental Safety	−0.659	0.204	−0.380	0.002*	−1.066	−0.252
Quality of Supervision	−0.169	0.148	−0.122	0.258	−0.464	0.126
Co‐worker Support	0.242	0.255	0.117	0.346	−0.266	0.751

*Note:* Multiple Regression analysing the following predictors: Perception of Sex Offenders Scale (PSOS), Attitudes Towards Sex Offenders (ATS‐21), Quality of Work Life Survey (QWLS).

* *p* < 0.05.

### 3.3 Burnout

The third regression model analysed predictors of burnout and was significant (*F* (9, 81) = 16.750, *p* < 0.001), explaining 65% of the variance in burnout.

#### 3.3.1 Perceptions and Attitudes

Results showed that several factors significantly predicted burnout. Negative perceptions of sentencing management (PSO Sentencing Management) were associated with lower burnout levels (*β* = −0.400, *p* < 0.001), suggesting that holding a more punitive stance towards sex offenders may reduce feelings of burnout. Similarly, cognitive evaluations of sex offenders (ATS‐21 Intent) were linked to lower burnout (*β* = −0.867, *p* < 0.001), highlighting that a more positive perspective on sex offenders can help to decrease burnout risk.

#### 3.3.2 Workplace Factors

Workplace factors were also influential. Higher environmental safety predicted lower burnout levels (*β* = −0.783, *p* < 0.001), underscoring the importance of a safe work environment in preventing burnout. Quality of supervision also negatively predicted burnout (*β* = −0.347, *p* = 0.003), emphasising the protective role of high‐quality supervision against burnout (Table 4).

**TABLE 4:** Psychological and workplace variables predict burnout.

Variable	Unstandardized coefficients		Standardised coefficients beta	Sig.	95% CI	
	*B*	Std. error			Lower bound	Upper bound
*PSOS*						
Sentencing Management	−0.400	0.080	−0.892	< 0.001*	−0.559	−0.241
Stereotype Endorsement	0.062	0.115	0.058	0.594	−0.167	0.291
Risk Perception	0.072	0.120	0.061	0.550	−0.166	0.310
*ATS‐21*						
Trust	0.170	0.111	0.179	0.129	−0.050	0.391
Social Distancing	−0.211	0.136	−0.203	0.123	−0.481	0.059
Intent	−0.867	0.124	−1.097	< 0.001*	−1.112	−0.621
*QWLS*						
Environmental Safety	−0.783	0.155	−0.521	< 0.001*	−1.091	−0.475
Quality of Supervision	−0.347	0.112	−0.289	0.003*	−0.570	−0.123
Co‐worker Support	0.337	0.194	0.187	0.086	−0.049	0.722

*Note:* Multiple Regression analysing the following predictors: Perception of Sex Offenders Scale (PSOS), Attitudes Towards Sex Offenders (ATS‐21), Quality of Work Life Survey (QWLS).

* *p* < 0.05.

## 4 Discussion

The findings of this study underscore the interplay between attitudes, perceptions and workplace factors in shaping the well‐being of forensic healthcare workers who work directly with sex offenders. For MHNs, who collaborate with and supervise HCAs, these findings emphasise their role in fostering team resilience and improving care delivery. Rational attitudes, such as understanding offenders' motivations, were linked to better well‐being, while certain negative perceptions, like punitive approaches, acted as protective mechanisms. MHNs can guide HCAs in balancing these perceptions with evidence‐based practices, promoting both resilience and therapeutic outcomes.

Workplace factors, particularly environmental safety and quality supervision, were critical in reducing burnout and stress. These findings highlight MHNs' responsibility to advocate for safer environments and provide structured supervision to support HCAs effectively. This study provides a basis for practical recommendations to strengthen MHN‐HCA collaboration and improve staff and patient outcomes in forensic mental health settings.

We extend existing research by focusing on the attitudes and perceptions of frontline, patient‐facing forensic healthcare workers towards sex offenders, an often‐overlooked group. Key findings revealed unexpected patterns, such as differing predictive effects of ATS‐21 subscales and PSO measures on workplace well‐being. Attitudes, involving emotional, cognitive, and behavioural elements and perceptions, reflecting broader judgements, are distinct yet complementary (Cacioppo et al. 1999), supporting Harper and Hogue's (2015) view of the ATS‐21 and PSO as complementary tools.

Positive attitudes like rational cognitive explanations of offenders' motivations and behaviours (ATS‐21 Intent) reduce stress and burnout and enhance compassion satisfaction. Conversely, negative attitudes like the desired level of social distance respondents are comfortable with (ATS‐21 Social Distancing) were linked to higher compassion satisfaction, highlighting the protective role of boundaries. Conversely, the level of trust respondents have towards sex offenders (ATS‐21 Trust) was associated with reduced compassion satisfaction, emphasising the complexity of balancing trust and boundaries.

The *Intent* attitude stands out as the most beneficial for both workers well‐being and therapeutic outcomes. Our findings link higher levels of this attitude to improved well‐being, while evidence highlights its role in fostering effective therapeutic environments and supporting offender reintegration (Hogue and Harper 2019). Similarly, the Good Lives Model advocates for understanding offenders' motivations, enhancing both rehabilitation outcomes and therapeutic engagement (Ward et al. 2007). Nurturing this attitude is therefore essential for practitioner well‐being and successful client recovery.

On the other hand, negative perceptions, such as support for punitive measures, restrictive management and the belief in a high likelihood of reoffending, can serve as protective psychological buffers for professionals by providing a sense of control and safety. However, these perceptions can also have a detrimental impact on the therapeutic climate, as they are often associated with reduced rehabilitation outcomes and strained professional‐offender relationships. Research shows that reoffending rates for sex offenders are significantly lower than widely believed, with many studies indicating rates of sexual recidivism below 20% over a 10‐year period, depending on offender characteristics and interventions (Hanson and Morton‐Bourgon 2019). Despite this, sex offenders often face highly restrictive sentences, including requirements to disclose their location if placed in the community, stringent monitoring and registration on offender lists.

Bearing in mind these realities, there is a strong case for emphasising attitudes such as *Intent*, which promote fairer and more constructive approaches to sentence management and risk perception. Shifting focus from solely punitive measures to understanding motivations and behaviours can help create a therapeutic environment that encourages change while addressing public safety concerns. Training professionals to build this understanding may not only enhance offender outcomes but also support practitioners' well‐being, balancing the need for protective mechanisms with fostering effective rehabilitation.

Workplace factors examined in this study aligned with findings from previous research (Ennis and Horne 2003; Hatcher and Noakes 2010). Environmental safety emerged as a strong positive predictor of all three components of workplace well‐being, highlighting the critical role of safety measures, particularly in high‐risk inpatient settings where relational dynamics pose significant challenges (Hatcher and Noakes 2010; Mivshek and Schriver 2022). Quality supervision also predicted reduced burnout, reinforcing its established role as a crucial coping mechanism for healthcare professionals. Supervision provides a structured space for debriefing, resilience‐building and mitigating stress (Hatcher and Noakes 2010; White et al. 2008). Maintaining professional boundaries is also essential in forensic settings, as boundary violations can compromise team cohesion and safety. Structured supervision and training are crucial in supporting staff to manage these risks effectively.

Unexpectedly, co‐worker support negatively predicted well‐being by reducing compassion satisfaction, challenging the commonly held view that peer support universally reduces psychological distress (Ennis and Horne 2003). This finding suggests that co‐worker interactions may sometimes amplify stress, potentially due to shared frustrations or a lack of professional boundaries. Evidence supports this interpretation, as studies on peer support workers highlight challenges such as emotional contagion, shared trauma and the cumulative impact of hearing multiple traumatic stories, which can exacerbate compassion fatigue (Steenekamp and Barker 2024).

These results underscore the importance of prioritising environmental safety and high‐quality supervision to enhance workplace well‐being and, by extension, the quality of care provided to sex offenders. Such measures not only support practitioners' mental health but also create a more stable and effective therapeutic environment (Delgadillo et al. 2018; Salyers et al. 2015; Teoh et al. 2023). As supported by Hatcher and Noakes (2010) and White et al. (2008), MHNs play a critical role in providing structured supervision and fostering a supportive workplace culture. Such leadership approaches strengthen workplace resilience and enhance the support available to frontline staff in demanding forensic settings.

### 4.1 Limitations and Strengths

The researcher's personal experience within privatised settings may have influenced the study design and interpretation of findings. However, this bias was mitigated through the supervision process. Future research should include NHS‐employed workers to identify potential implications for non‐private healthcare workplace well‐being. Nonetheless, the study was adequately powered, and the measures included in the study achieved good internal consistency and reliability, highlighting the potential generalisability of the findings. Another limitation is the uncertainty around the influence of participants' contact time with non‐sex offender clients on workplace well‐being. Forensic mental health staff typically support individuals with various offences, and exposure to clients without sexual offences may have influenced the data. However, the study's measures focused exclusively on attitudes and perceptions towards sex offenders, strengthening the methodology's validity. The average participant had 2 years and 5 months of experience working with sex offenders, further ensuring the well‐being results reflect their specific experiences. An additional limitation is that, while the sample included variation in participant characteristics such as sex, age, nationality and experience, subgroup analyses were not conducted due to the limited sample size. This constraint means potential differences between these groups remain unexplored, and future research with larger samples should examine these aspects to provide a more nuanced understanding of workplace well‐being.

## 5 Conclusion

In summary, the findings provide valuable insights into the predictors of well‐being among forensic healthcare workers, such as HCAs and nurses who work directly with sex offenders and did not receive training on attitudes towards sex offenders. The findings highlight the importance of considering attitudes and perceptions as distinct psychological processes with differing effects on well‐being. For MHNs, these insights underline their critical role in guiding HCAs to adopt rational attitudes that support professional boundaries, maintain emotional distance and foster balanced approaches to offender management. Ensuring adequate levels of environmental safety and supervision remains essential for fostering a healthier workforce. By prioritising MHN‐led supervision and collaborative training initiatives, employers can enhance staff resilience and improve therapeutic outcomes, benefiting both carers and service users. Frontline staff, with their extensive patient contact, are critical to daily care in forensic settings. Extending workplace supports such as supervision and environmental safety to these staff members is essential for sustaining effective care delivery.

## 6 Recommendations

The authors highlight the importance of fostering rational attitudes, such as understanding offenders' motivations, to improve staff well‐being and therapeutic outcomes. For MHNs, this includes mentoring HCAs in adopting these attitudes through supervision and joint training initiatives. Environmental safety and high‐quality supervision are essential, with employers encouraged to implement robust safety measures and structured debriefing sessions. MHNs should lead efforts to address peer dynamics, ensuring that co‐worker interactions remain supportive and do not contribute to stress or reduced compassion satisfaction. While punitive perceptions can provide emotional buffering, staff training should emphasise evidence‐based approaches that balance safety with effective rehabilitation practices. By equipping MHNs to guide HCAs in balancing professional boundaries and therapeutic goals, employers can strengthen collaborative care and improve outcomes for both staff and patients.

## 7 Disclaimer

During the preparation of this manuscript, the author(s) used ChatGPT 4o (OpenAI 2024) in order to improve readability and condense the original thesis chapter. After using this tool/service, the final author reviewed and edited the content as needed and took full responsibility for the content of the publication. This process provides a time‐stamped record demonstrating that the main content was not created by AI.

## Conflicts of Interest

The authors declare no conflicts of interest.

## Data Availability

The data supporting the findings of this study are not publicly available.

## Appendix A

### STROBE Checklist Compliance for Cross‐Sectional Studies

**Table:**

STROBE item	Recommendation	Implementation in the study	Page no.
Title and Abstract	Indicate the study's design and provide a balanced summary of methods and findings	The title specifies that the study is cross‐sectional, providing clarity for readers. The abstract includes a concise description of the study objectives, methods (e.g., use of validated scales like ProQOL and ATS‐21), key findings and implications	1–2
Introduction	Explain scientific background, rationale and objectives	The background outlines the importance of studying well‐being in frontline forensic healthcare workers, particularly those working with sex offenders, highlighting gaps in prior research. Objectives are clearly stated, including hypotheses about predictors of well‐being	3–7
Methods	Present design, setting, participants, variables, data sources and statistical methods	The methods section describes the cross‐sectional design, recruitment settings (UK‐based privatised mental health facilities), eligibility criteria (e.g., frontline workers with direct contact with sex offenders and no training focused on attitudes; HCAs, nurses), variables (e.g., compassion satisfaction, burnout) and statistical methods. G*Power software was used to ensure adequate power. Incomplete responses (*N* = 40) were excluded from analysis leaving final *N* = 91	8–11
Statistical Methods	Describe all statistical methods, including those used to control for confounding	Three multiple regression analyses were conducted to examine predictive effects among variables, with each ProQOL subscale (compassion satisfaction, burnout and secondary traumatic stress) serving as the dependent variable. Predictors included PSO subscales, ATS‐21 subscales and workplace variables (environmental safety, perceived peer support and quality of supervision). Data were exported from Qualtrics into IBM SPSS Version 27.0 for analysis	11
Bias	Describe efforts to minimise potential bias	Bias was addressed by using validated measurement tools (ProQOL, ATS‐21 and PSO) to ensure reliability and validity. Standardised procedures for data collection were implemented to minimise variability	8–10
Results	Report participant characteristics, outcome measures and key results	Participant demographics (e.g., age, gender, experience), professional backgrounds status and attitudes training status were reported. Outcome measures for well‐being, burnout, and secondary traumatic stress were detailed and results were presented with appropriate statistical summaries (e.g., regression coefficients, confidence intervals)	12–20
Discussion	Summarise key results, address limitations and discuss generalisability	Key results are linked to the study's objectives, emphasising the role of attitudes, perceptions and workplace factors in shaping well‐being. Limitations (e.g., focus on privatised settings) are discussed, and the generalisability of findings to other healthcare contexts is considered	21–25
Funding	Provide funding details and the role of funders	The funding source is acknowledged, noting that funders had no role in study design, data collection, analysis, or interpretation of findings	1
